# Supplementary figures and images for: Brain signal predictions from multi-scale networks using a linearized framework
Source: PLoS Comput Biol. 2022 Aug 12;18(8):e1010353. doi: 10.1371/journal.pcbi.1010353 (PMC9401172; doi:10.1371/journal.pcbi.1010353)

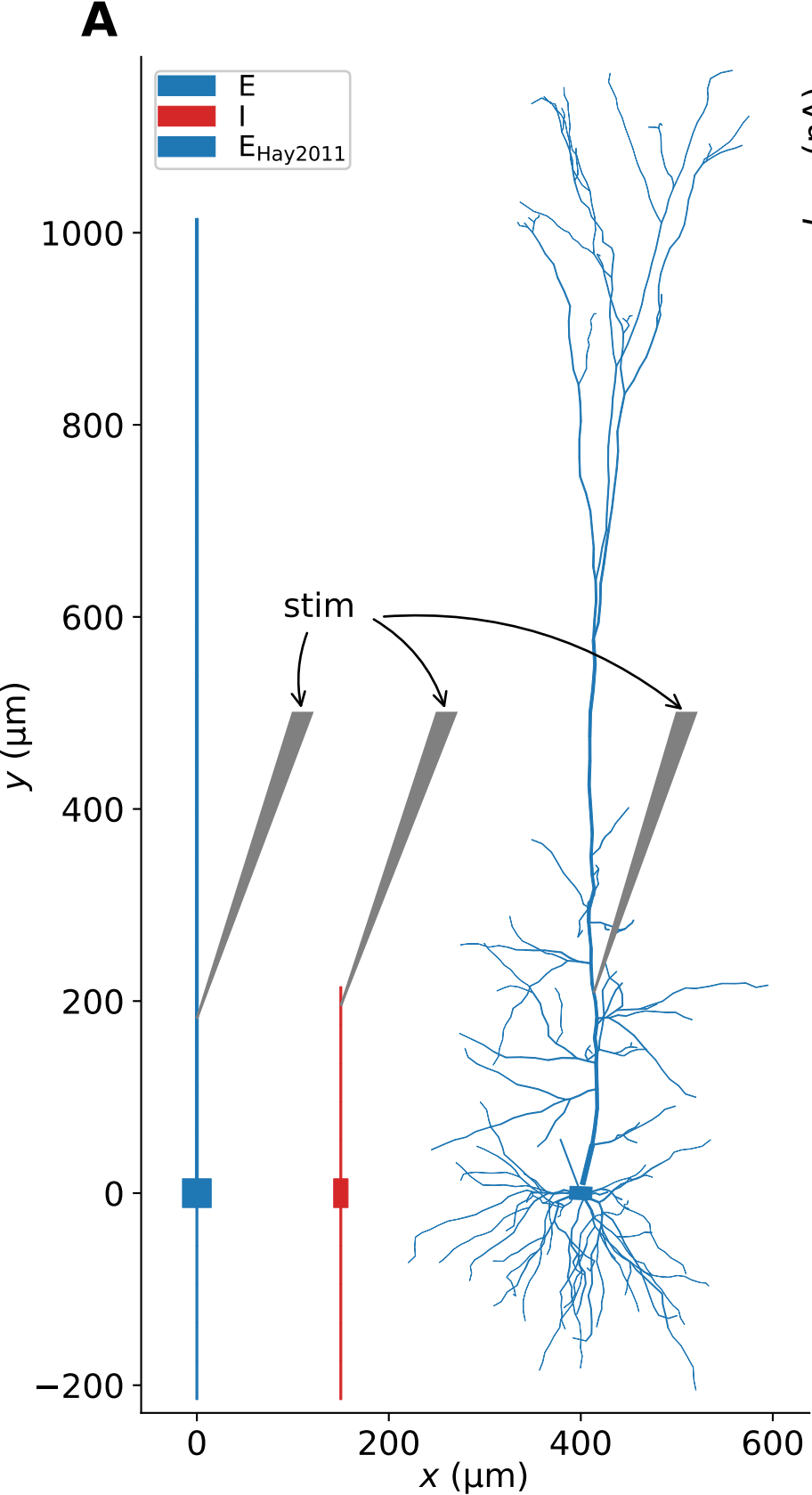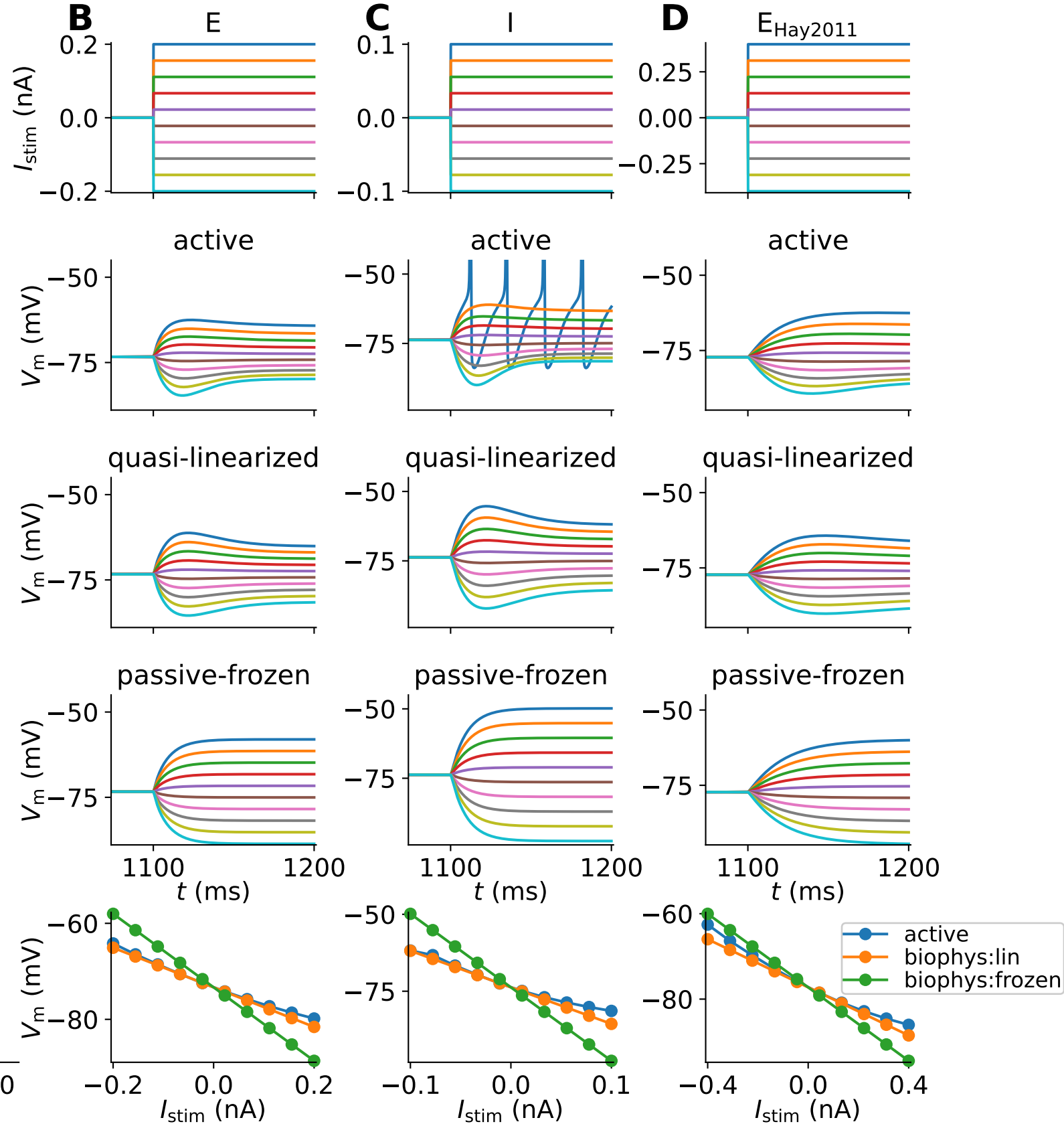

Supplement: S1 Fig — Same as Fig 2, but with current input in the apical dendrites approximately 200 μm from the soma compartments of the respective neurons. (PDF) [file pcbi.1010353.s001.pdf]

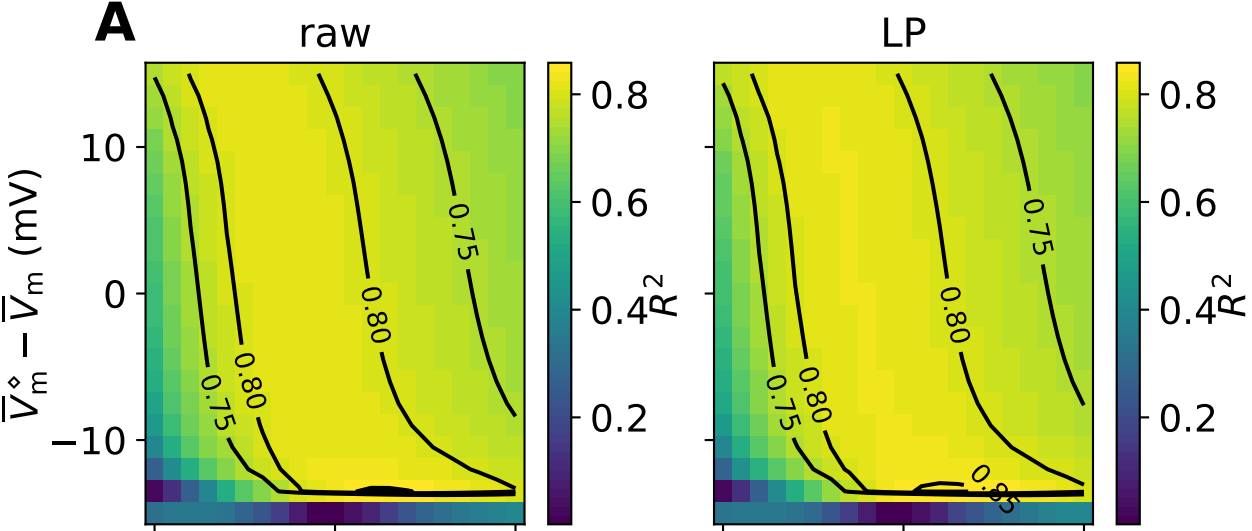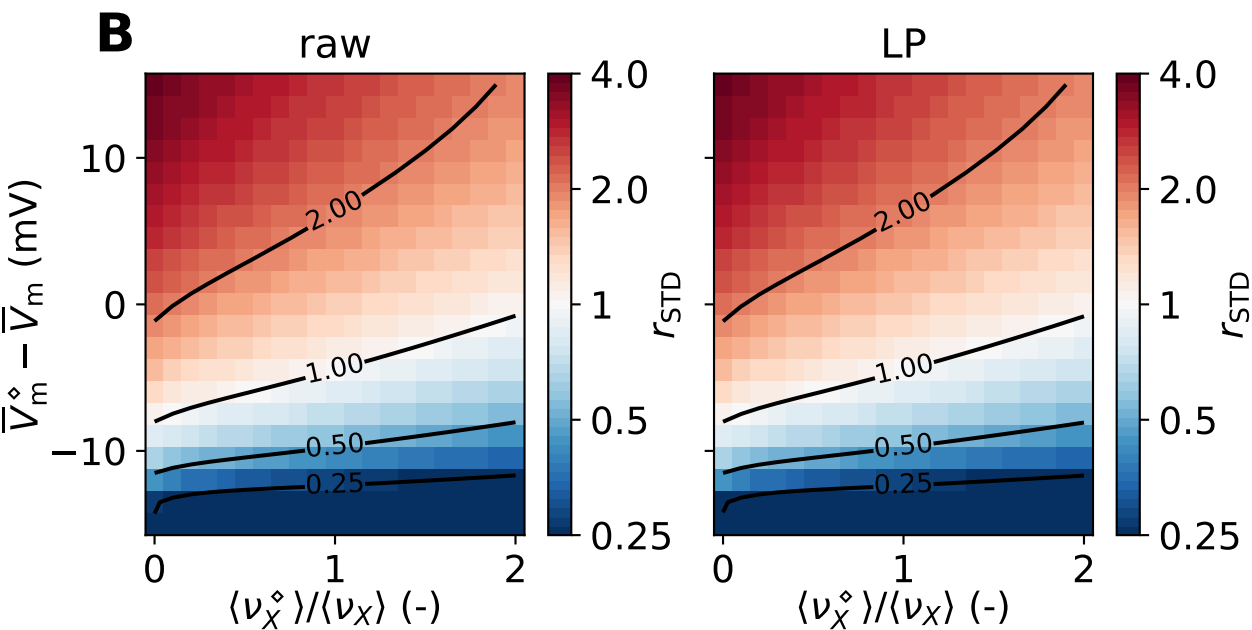

Supplement: S2 Fig — Same as Fig 8, but for the case where the excitatory (‘E’) population is replaced by biophysically detailed neuron models [30]. (PDF) [file pcbi.1010353.s002.pdf]

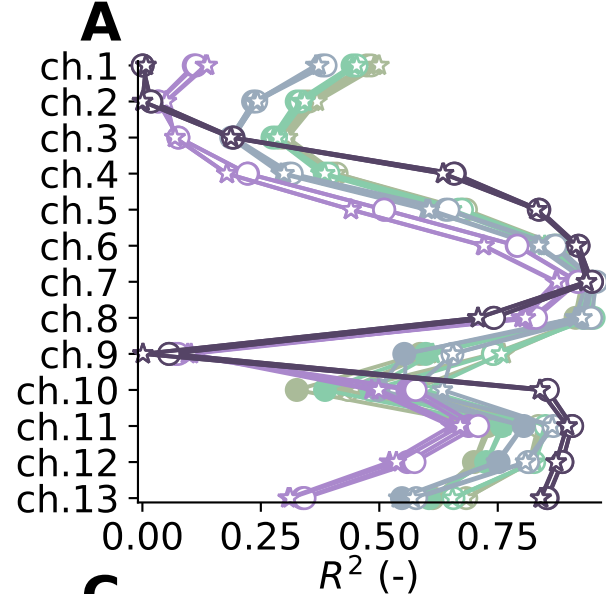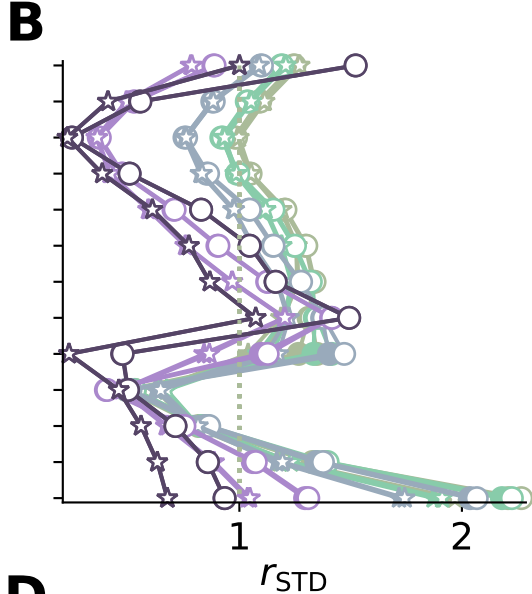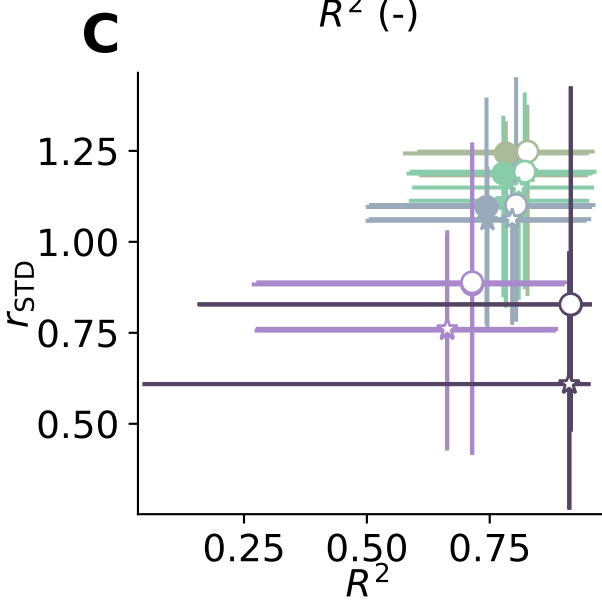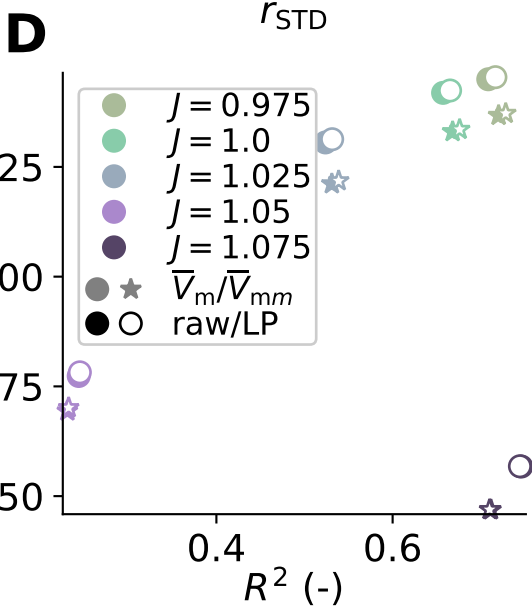

Supplement: S3 Fig — Same as Fig 13C–13F, but for computed kernels and reconstructed signals either assuming a constant value for V¯m across the entire neuron model (circular lines/markers), versus kernel-based predictions where the V¯m is set on a per-compartment basis (asterisk markers). For this, we use averaged values from each reference network simulation providing ground truth signals for comparison. Same color coding as in Fig 13. (PDF) [file pcbi.1010353.s003.pdf]
